# The injections of mitochondrial fusion promoter M1 during proestrus disrupt the progesterone secretion and the estrous cycle in the mouse

**DOI:** 10.1038/s41598-023-29608-7

**Published:** 2023-02-10

**Authors:** Yovita Permata Budi, Meng-Chieh Hsu, Yi-Chun Lin, Yue-Jia Lee, Hsin-Yi Chiu, Chih-Hsien Chiu, Yi-Fan Jiang

**Affiliations:** 1 Graduate Institute of Molecular and Comparative Pathobiology, School of Veterinary Medicine, National Taiwan University, Rm. 104-1, No.1, Sec. 4, Roosevelt Road, Taipei, 10617 Taiwan; 2 School of Veterinary Medicine, National Taiwan University, Taipei, 10617 Taiwan; 3 Department of Animal Science and Technology, National Taiwan University, Taipei, 10617 Taiwan; 4 Department of Animal Science, National Chung Hsing University, Taichung, 40227 Taiwan; 5 Institute of Food Science and Technology, National Taiwan University, Taipei, 10617 Taiwan; 6 Division of Thoracic Surgery, Department of Surgery, Taipei Medical University Hospital, Taipei, 11031 Taiwan; 7 Department of Medical Education, Taipei Medical University Hospital, Taipei, 11031 Taiwan; 8 Department of Education and Humanities in Medicine, School of Medicine, Taipei Medical University, Taipei, 11031 Taiwan; 9 Department of Surgery, School of Medicine, Taipei Medical University, Taipei, 11031 Taiwan

**Keywords:** Physiology, Reproductive biology, Mitochondria

## Abstract

After ovulation, the mitochondrial enzyme CYP11A1 cleavage the cholesterol into pregnenolone for progesterone synthesis, suggesting that mitochondrial dynamics play a vital role in the female reproductive system. The changes in the mitochondria dynamics throughout the ovarian cycle have been reported in literature, but the correlation to its role in the ovarian cycle remains unclear. In this study, mitochondrial fusion promotor, M1, was used to study the impact of mitochondria dynamics in the female reproductive system. Our results showed that M1 treatment in mice can lead to the disruptions of estrous cycles in vagina smears. The decrease in serum LH was recorded in the animal. And the inhibitions of progesterone secretion and ovulations were observed in ovarian culture. Although no significant changes in mitochondrial networks were observed in the ovaries, significant up-regulation of mitochondrial respiratory complexes was revealed in M1 treatments through transcriptomic analysis. In contrast to the estrogen and steroid biosynthesis up-regulated in M1, the molecules of extracellular matrix, remodeling enzymes, and adhesion signalings were decreased. Collectively, our study provides novel targets to regulate the ovarian cycles through the mitochondria. However, more studies are still necessary to provide the functional connections between mitochondria and the female reproductive systems.

## Introduction

Mitochondria are dynamic organelles. In addition to continually fusing and dividing, they are recruited to specific locations within cells^1^. With their active structure, the mitochondria maintain their shape, distribution, and size by undergoing coordinated cycles of fission and fusion, referred to as mitochondrial dynamics. Fission and fusion are active processes that require many specialized proteins, including mechanical enzymes that physically alter mitochondrial membranes and adaptor proteins that regulate the interaction of these mechanical proteins with organelles^1^. The fusion increases mitochondrial volume for higher energy production, while the fission removes the dysfunctional population that contains damaged protein, membrane, or mtDNA. The dynamics thus serve as the quality and quantity control mechanism for intracellular mitochondrial populations^2^. The balanced dynamic transitions are required to ensure mitochondrial function and respond to cellular needs by adapting the network to nutrient availability and to the cell's metabolic state under physiological and pathophysiological conditions^3^.

Mitochondria could also play a critical role in female reproduction. Granulosa cells and oocyte mitochondria have been linked to oocyte quality and ovarian aging^4^. Also, there has been evidence of mitochondrial biogenesis in goats during follicular development^5^. In luteal formations, as the granulosa cells proliferate and differentiate in corpus hemorrhagicum, changes in mitochondrial structure were observed in our previous study^6^. In mice, the estrous cycle lasts 4–5 days and could be divided into proestrus, estrus, metestrus, and diestrus by the cytological examinations of vagina smears^7^. Generally, ovulation could occur in the morning of estrus, after the luteinizing hormone (LH) surge in the late afternoon of the proestrus. In contrast to the elevation of estradiol before the LH surge, an elevating progesterone level could be recorded on the evening of proestrus^8^. The impacts of mitochondrial dynamics on steroidogenic cells have been studied in various models, including Leydig cells and luteal cells^9,10^. The defects in the mitochondrial fusion protein, Mitoguardin-1/2, also show a subfertile phenotype in female mice^11^. However, more studies are still necessary to clarify the relationships between mitochondrial dynamics and ovarian cycles.

The mitochondrial fusion promoter M1 is a cell-permeable phenylhydrazone compound that restores tubular network formation in various tissues and cells, including mouse embryonic fibroblasts^12^, human iPSCs^13^, rat cardiomyocytes^14^, PC12 cells^15^, isolated mouse T cells^16^, neurons^17^, and brain tissues^18^. An increase in the mitochondrial spare respiratory capacity, oxygen consumption rate, and genes of respiratory complexes has also been reported after M1 treatments^12,14,16^. Considering the lack of understanding of the role M1 plays in ovarian cycles and the role of mitochondrial dynamics, the current study is aimed at investigating the effects of mitochondrial fusion promoter M1 on mouse ovarian functions, using the M1 fusion promoter to manipulate mitochondria. The ovarian functions after M1 treatments were checked through vagina smear and RNA sequencing in adult female mice. While the 3D mitochondrial networks were checked at ultrastructural levels, the direct impacts of M1 on the ovary were studied through ex vivo ovarian cultures.

## Results

### The impact of M1 on the estrous cycle

**Figure 1:**
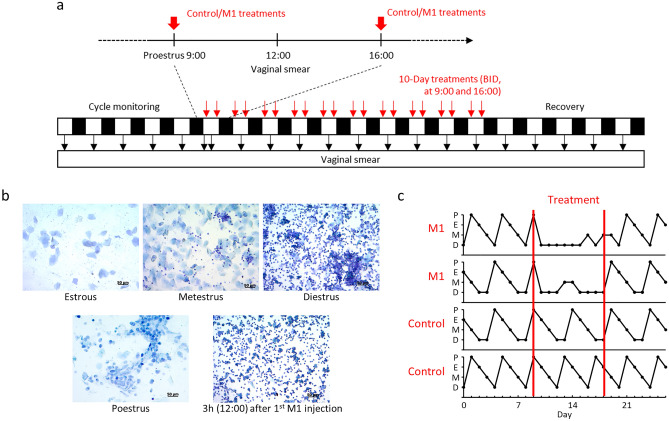
The proestrus M1 injections temporarily disrupted the mouse estrous cycle. A group of 8-week-old ICR mice estrous cycle was observed by vaginal smear for cycle monitoring. After two or more cycles were observed, the mice were treated with M1 or Control treatments (n = 4) twice a day for 10 consecutive days and the mice were then observed to recover (**a**). The figure shows the estrus cycle of the mice from before, during, and after the treatments (**b**). The estrus cycle of the mice was observed by vaginal smears and the comparison of the representative smears 3 h after the first M1 injection was shown (**c**).

To test if the M1 can impact ovarian functions, the estrous cycles of 8-week-old ICR mice were monitored through daily vaginal smears. After stable cycles were observed, the mice on proestrus were treated twice a day with M1 for 10 days followed by a recovery period without injections. Observations on the changes in estrus cycles proceeded from the vaginal smear (Fig. 1a). In mice treated with M1 in the proestrus morning (9:00), the cycle disruptions were observed right at noon (12:00), when the signs of diestrous were shown in the smear (Fig. 1b). As the injections continued, the M1-induced diestrous phase was elongated but the cycles were immediately recovered after treatment was stopped (Fig. 1c).

### The impact of M1 treatments on the reproductive endocrine system

**Figure 2:**
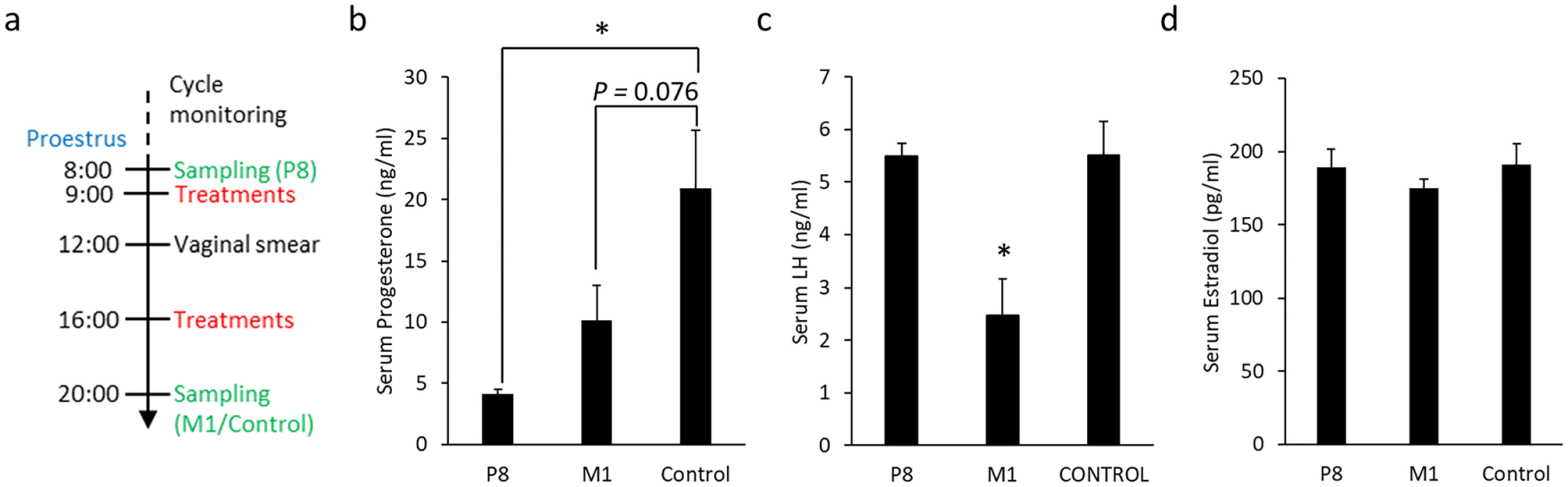
The impacts of M1 injection on the endocrine system. Eight-week-old ICR mice were treated with M1 at 9:00 and 16:00 on the day of proestrus, the blood samples were collected in the evening (20:00, **a**). The concentrations of serum progesterone (**b**), LH (**c**), and estradiol (**d**) were measured. Values are mean ± SEM (n = 4–7). *Statistical significance by Student's t-test, *P* < 0.05.

The endocrine level during the proestrus phase in mice is more fluctuated compared to the other phases. There are contrasting events that happen during the light and dark periods of the proestrus phase^8^. Considering the sufficient time to obtain significant changes in the transcriptome and hormone analysis within the same stage, blood samples were collected on the proestrus evening (20:00) after the second injections of the day (M1 and Control group, Fig. 2a). To demonstrate the changes in hormone concentrations, the blood samples were also collected on the morning (8:00) of proestrus (P8). By the sampling time, the serum progesterone level of the control group was higher than the P8 group (Fig. 2b) while the serum LH of the M1 group was lower than the others (Fig. 2c). Additionally, there were no significant differences between the groups in serum estradiol levels (Fig. 2d). Based on the higher variations in serum progesterone levels, the mean value was lower than the control value but did not reach statistical significance (*P* = 0.076, Fig. 2b). In this study, attempts were also made to check the serum FSH level. However, the FSH level in serum were all under the detection limit of the available ELISA system (data not shown).

### The mitochondrial network in the ovaries

**Figure 3:**
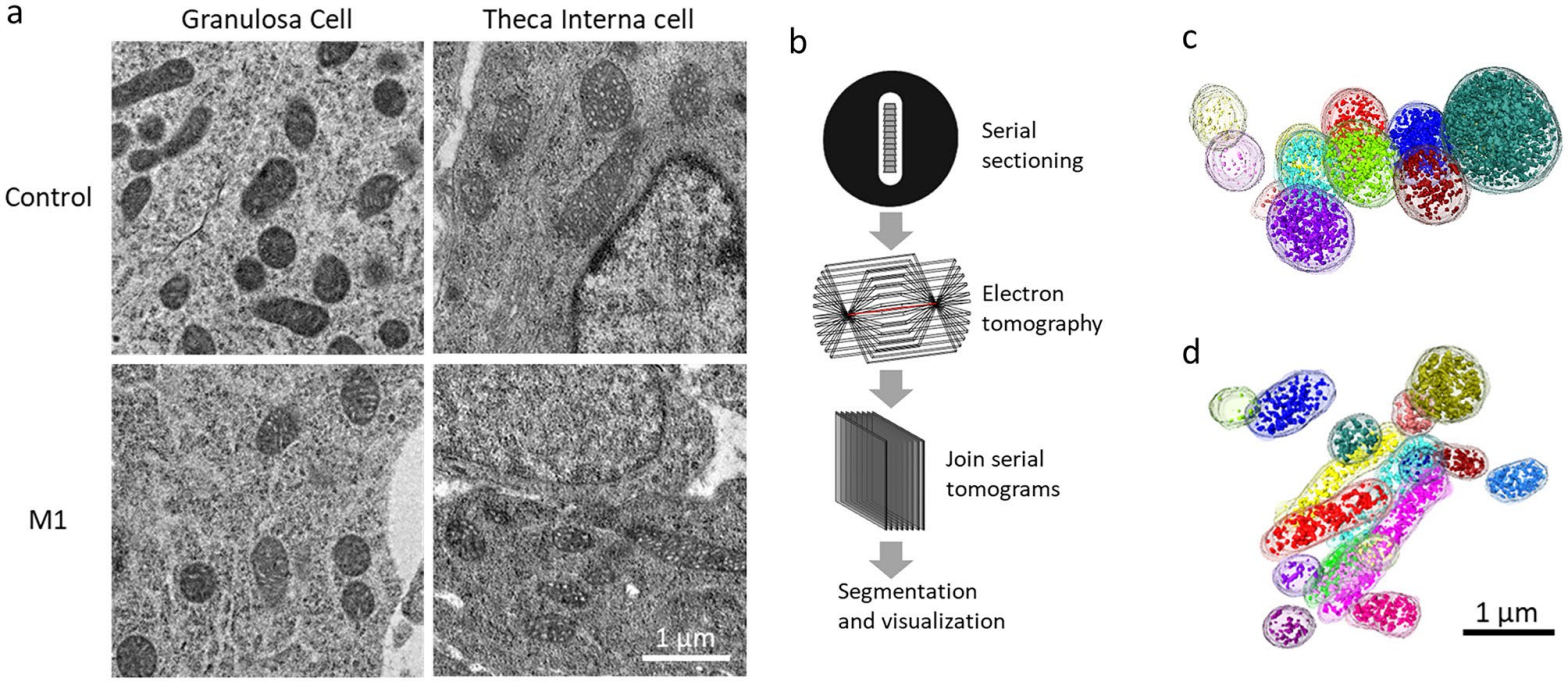
M1 injection did not change the mitochondrial network of the granulosa cells and theca cells in matured follicles. The samples were collected in the evening (20:00) of proestrus after the second M1 or control injections. The electron micrograph shows the rod- or globular-shaped mitochondria in both granulosa cells and theca cells (**a**). The flowchart for the visualizations of 3D mitochondrial networks (**b**). The 3D mitochondrial networks in control (**c**) and M1 (**d**) theca cells were reconstructed through the serial section and electron tomography. The mitochondrial cristae were labeled in opaque colors and the outer membranes were labeled in transparent colors. The mitochondria without physical connections were labeled with different colors. No obvious fusing or branch mitochondria were observed.

Electron microscopy was used to observe and compare mitochondria in the ovary after the M1 treatment. For each group, the mitochondria in granulosa and theca cells were screened through 2D images (Fig. 3a). The mitochondria in granulosa cells showed lamellar cristae, whereas the mitochondria in theca cells showed tubular or vesicular cristae. The mitochondria in rod- or globular-shape were observed in both types of cells. No significant changes in mitochondria structure were noted in the M1 group (Fig. 3a). To confirm the possibility of the 3D networks in the cells, serial section and electron tomography were performed (Fig. 3b). In our investigation, no hyper-fused nor branched mitochondrial networks were observed (Fig. 3c,d, and Movie S1–S4).

### The gene expression

**Figure 4:**
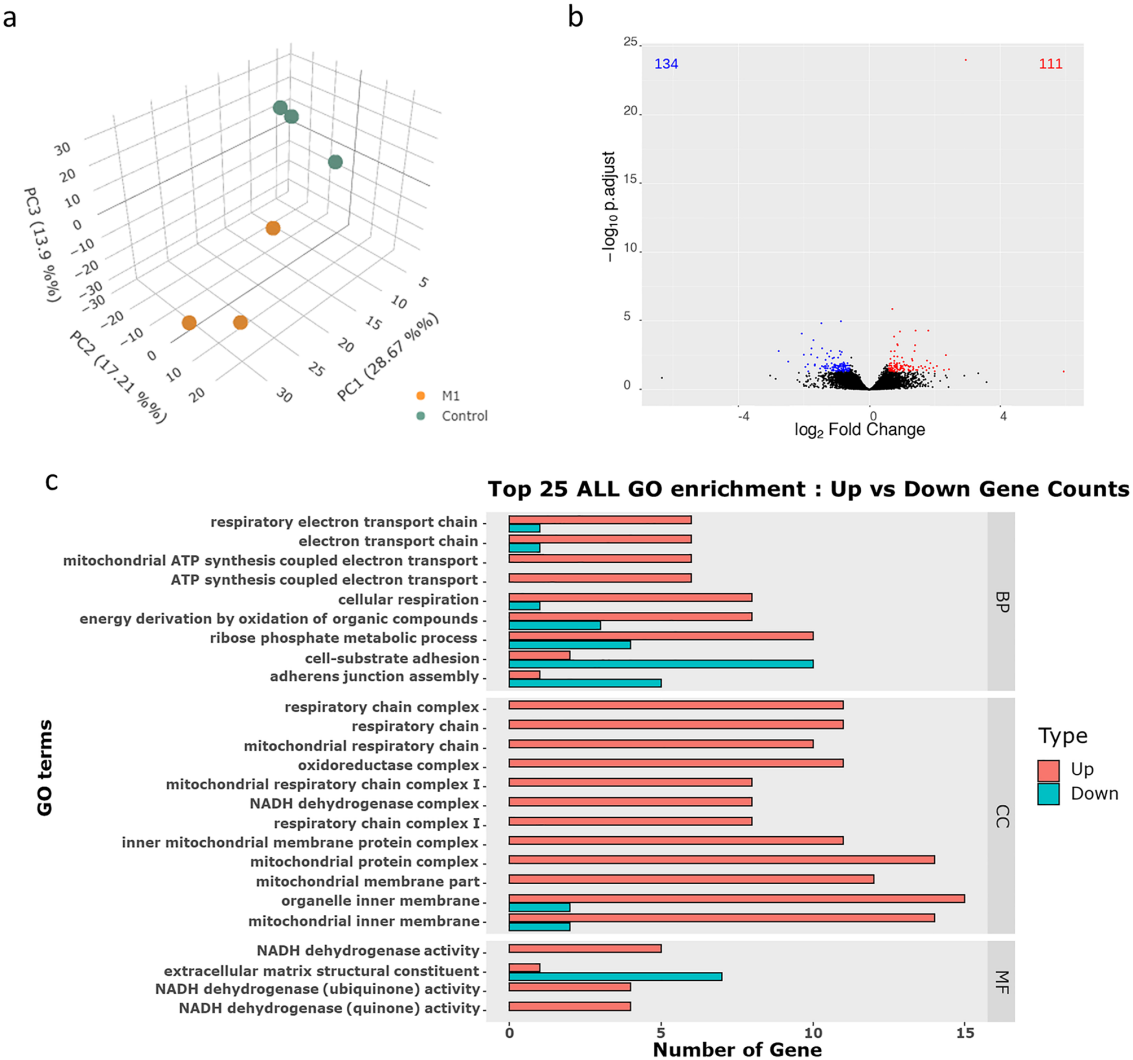
M1 injection promoted the expression of the genes of mitochondrial respiratory chain complexes and suppressed the genes associated with cell adhesion in the ovary. After M1 or control treatments on proestrus, the ovaries were collected in the evening (20:00) for transcriptome analysis (n = 3). The 3D principal component analysis revealed the similarity among samples and groups (**a**). The volcano plot showed the distribution of differentially expressed genes with *P-*adjust values and fold changes (**b**). The genes were filtered and GO analysis was performed, top 25 GO terms (*P* < 0.05) were shown in the compare barplot (**c**).

To clarify the mechanism of how M1 might disrupt the estrus cycle, transcriptome analysis was performed on the ovary after the M1 treatment on the day of proestrus (20:00). In totally 6 submitted samples, an average of 41.7 million reads were obtained, where 97.02% of the reads were successfully mapped (90.68% uniquely mapped, Table S1). 14,645 differentially expressed genes (DEGs) were obtained and listed (Table S2). The principal component analysis (PCA) between M1 and Control treatments revealed a clustered distribution within the groups, while the variations in biological repeats could also be noted (Fig. 4a).

The DEGs were filtered for GO and KEGG pathway analysis, and with our settings, a total of 245 DEGs were obtained (134 down-regulated and 111 up-regulated in the M1 group, Fig. 4b). In GO analysis, 41 significant enrichment terms (adjust* P* value < 0.05) were revealed, including 12 in cellular component (CC), 6 in molecular function (MF), and 23 in biological process (BP, Table S3). 29 of the significant enrichment terms in our comparison were associated with the mitochondrial respiratory chain, where the members of the genes were up-regulated in the M1 group, suggesting the specific effects of M1 on ovarian mitochondria (Fig. 4c). On the other hand, the down-regulated genes in the M1 group could be found in cell-substrate adhesion, adherens junction assembly, and extracellular matrix (ECM) structural constituent (Fig. 4c). Similar results could also be found in the KEGG pathway analysis (Table S4). In 14 of the totally obtained 15 pathways, the genes in the mitochondrial respiratory chain and the signaling of focal adhesion were included. Interestingly, steroid biosynthesis was recognized as a significantly up-regulated pathway in the M1 group, where 3 genes could be obtained independently from the other pathways (Table S4).

**Figure 5:**
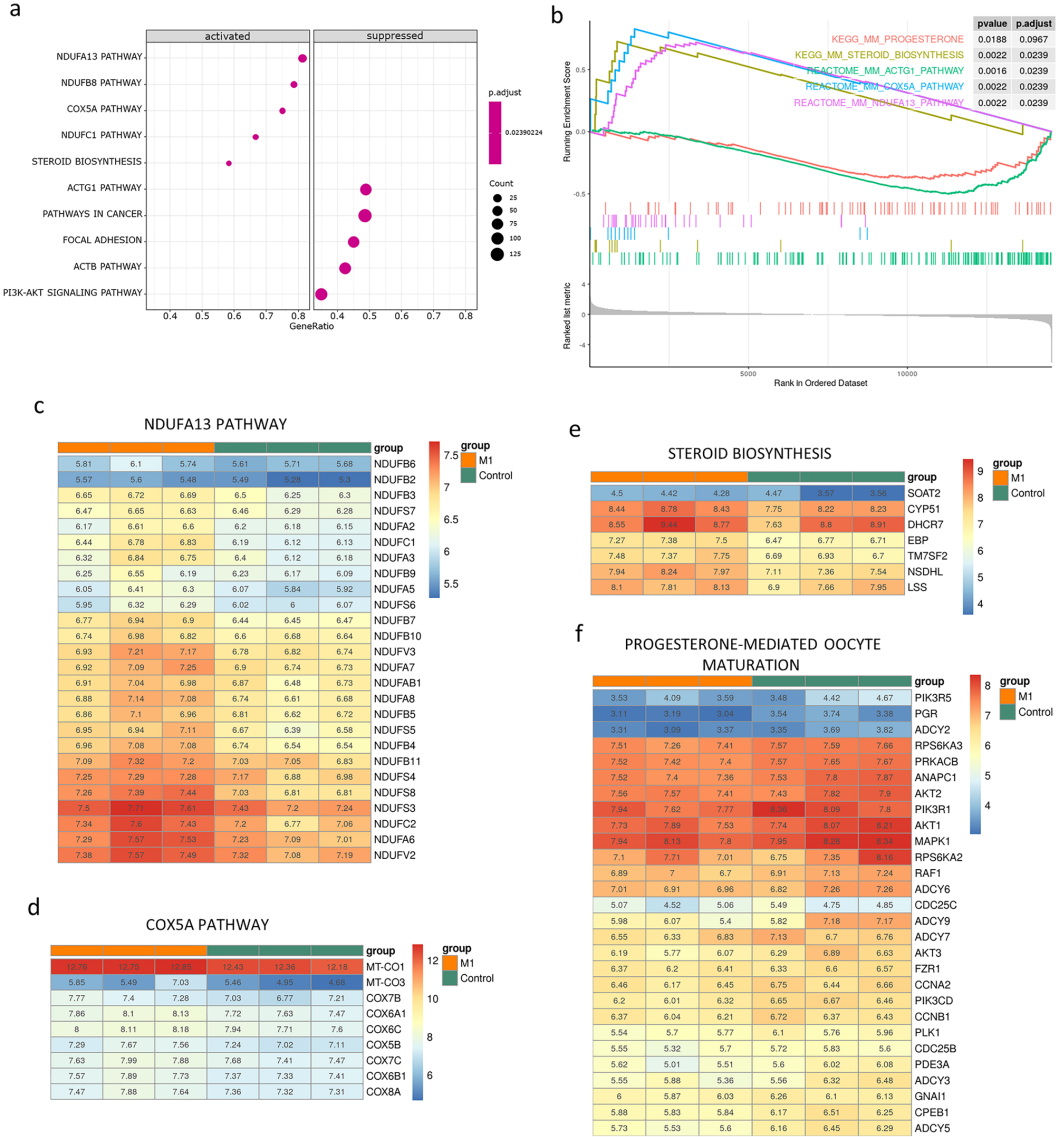
The significant metabolic pathways after M1 treatments were revealed in gene set enrichment analysis (GSEA). The genes identified as over-expressed were classified through the GSEA dot plot (**a**) and GSEA plot (**b**). The details of gene expressions for NDUFA13 (**c**) COX5A (**d**), steroid biosynthesis pathway heatmap (**e**), and progesterone-mediated oocyte maturation (**f**) were shown in heatmaps. The expression levels were normalized and shown as relative log expressions (RLE).

To investigate the gene expression in specific pathways, the gene set enrichment analysis (GSEA) was performed (Fig. 5). In 4604 analyzed gene sets from the database of the mouse metabolic pathways, 586 significant gene sets were obtained (adjust *P* < 0.25, Table S5). Similar to our post-filter GO and KEGG analysis, the top activated gene sets (M1 gene sets) included the members of NADH dehydrogenase (ex: NDUFA13, NDUFB8, and NDUFC1), Cytochrome C Oxidase (COX5A), and steroid biosynthesis (Fig. 5a). The members of top M1 gene sets showed a focused rank distribution in ordered datasets (Fig. 5b). The higher consistency of expression among individuals could be observed in the detailed heatmaps list (Fig. 5c–e). The suppressed gene sets (Control gene sets) were comparatively larger in our study, which could be reflected by higher gene count and lower gene ratio (Fig. 5a). The top control gene sets contained more overlapping members, including those for cell interactions, signaling, cytoskeleton, ECM molecules, and remodeling enzymes, which could be identified (Fig. 5b; Table S5; Fig. S1). The gene sets associated with progesterone-mediated oocyte maturation (KEGG pathway mmu04914) and estrogen synthesis were also obtained in our GSEA results (Fig. 5b; Fig. S2a). With higher false discovery rates (FDR, adjust *P* = 0.0967 and 0.1451, respectively), the progesterone gene sets were suppressed in M1 treatments (Fig. 5f), while the pathway of estrogen synthesis was activated in the M1 treatments (Fig. S2b).

### The ex vivo culture

**Figure 6:**
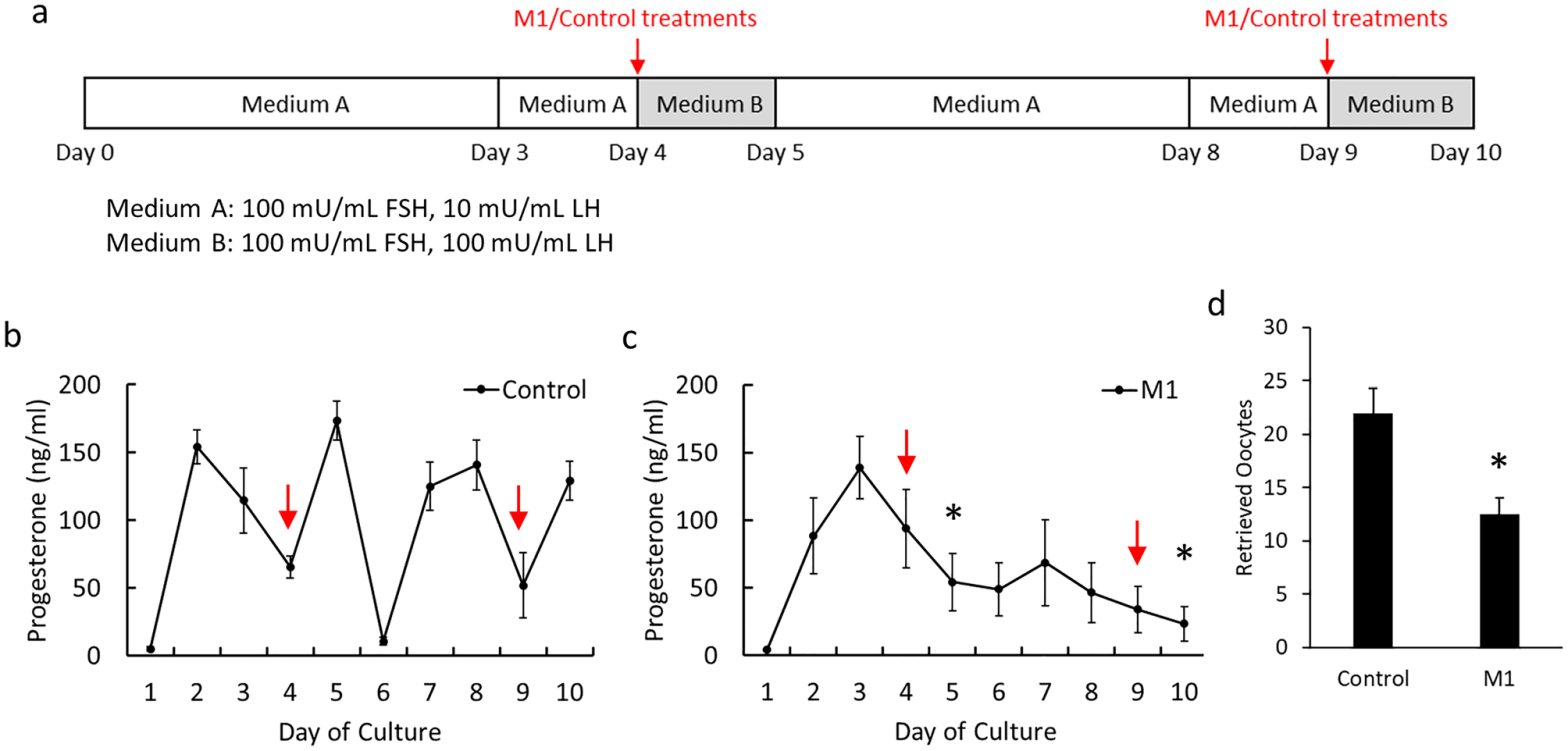
M1 suppressed progesterone secretion and oocyte release during LH stimulation in ovarian organ cultures. The mouse ovaries at proestrus were cultured in the medium containing FSH and LH to mimic the estrus cycle (**a**). The progesterone concentrations in the medium were analyzed in Control (**b**) and M1 groups (**c**). The number of oocytes retrieved from the ovarian culture was also counted (**d**). Values are mean ± SEM (n = 3–4 for each group). *Statistical significance when compared to the control on the same day, *P* < 0.05, Student's t-test.

The timing of the cycle could have higher variations and systemic complexities among individual animals. To simplify the factors and observe the direct impact of the M1 on the ovary, ovarian tissue cultures were performed. The treatments were delivered through the culture medium accompanied by consistent hormone levels (Fig. 6a). In order to manipulate mitochondrial dynamics during the transition between the follicular and luteal phases, M1 treatment and medium B (high LH) were administered simultaneously. During the culture periods, the culture medium was collected daily to access steroidogenic functions. The concentration of estradiol was below the detection limits in our system, so only the medium progesterone levels were presented (Fig. 6b,c). While the elevated progesterone level could be detected after the control LH stimulation on Day 5 and Day 10 (Fig. 6b), the combination of LH and M1 treatment resulted in a reduced medium progesterone secretion (Fig. 6c). In line with the reduction of the medium progesterone level, the number of retrieved oocytes from M1 treatment was also reduced (Fig. 6d).

## Discussion

The mitochondria in animal cells form a highly dynamic network under physiological conditions. Mitochondria continuously undergo biogenesis, fission, fusion, mitophagy, and motility^19^. Mitochondrial dynamics differ in different types of cells and meet the specific functional needs of the cell. Mitochondria are involved in apoptosis, ATP production, and steroidogenesis in the female reproduction system^20^. In goat large luteal cell lineage, significant mitochondria mass and shape changes have been noted during the transition from matured follicle to corpus luteum^6^. However, the impacts and regulatory mechanisms of mitochondrial dynamics in the ovarian cycle remain unsolved. In this work, we propose a novel mechanism by which the promotion of mitochondrial fusion may affect the ovarian cycle by altering mitochondrial function and steroid hormone production.

The regular reported length of the rodent estrous cycle is 4–5 days, which was also presented in the control group of our study^8,21^. The regular estrous cycle is an indicator of successive ovulation, cyclicity of which was found to be disrupted in the mouse during ten days of exposure to M1 and recovered after the treatment stop, suggesting the M1 could have a mild and reversible impact on the female reproductive system of the mouse. At the beginning of M1 treatments, the confirmed proestrus phase was changed to the diestrous phase 3 h after the first M1 injection, suggesting an acute response of the reproductive system to M1 exposure. The steroid hormones like estrogen and progesterone could regulate vaginal epithelial cell growth, leukocyte infiltration, and mucus secretions^7,22–24^. In rodents, the stage with the highest serum progesterone level has been reported in literature to be inconsistent, varying from late-proestrus, estrus, and diestrous^25–27^. This could be due to the variation and dramatic changes in reproductive events in rodents. In recent studies, the lowest progesterone concentration could be found in diestrus, while the elevation of progesterone happened in proestrus or estrus stage^25,26^. LH surge is also expected in the late proestrus, although the exact time window is hard to observe^28^. In our study, the progesterone was elevated in the proestrus evening, which is consistent with previous reports on rats^26^. The M1 injection resulted in an ambiguous reduction in serum progesterone, which could reflect the complexity of physiological events on the day of rodent proestrus. The LH showed a similar level between the proestrus morning and evening in the control group, suggesting the LH level could reflect the basal secretion level through our sampling schedule^8^. The LH was lower in the M1 group. Such changes could take part in the signaling of the ovarian cells. However, since the appearance of LH surge or ovulation is yet to be confirmed in vivo after M1 treatments, more sampling and observation remain necessary to evaluate the impacts of M1 in female mice. As was shown in our result, the endocrine inhibition in M1-treated mice might be similar to the hormone conditions in the diestrous stage and lead to the diestrous result in vaginal smear after M1 injections.

In our previous study, the elongated mitochondria with branches could be found in granulosa cells, broken down at luteinization in goats^6^. Yet the detailed timing for the shift of mitochondrial dynamics at ovulation remains unavailable. As increased mitochondrial fusion could lead to the formation of hyper-fused branching networks, we focused on the formation of mitochondrial networks in ovarian cells after M1 treatments. In mice, the mitochondrial networks observed in late proestrus already showed a fragmented pattern in the granulosa cells of the matured follicles. However, the stage definition of the cycle and cellular events could be inconsistent between rodents and other mammals. In theca interna cells, the elongated mitochondria were noted after M1 treatments in our 3D ultrastructural analysis. However, similar features could also be observed in the control group during 2D screening. Despite the fact that M1 seemed to promote mitochondrial fusion in various models^12–18^, the M1 injection did not change the mitochondrial network in the mouse granulosa cells and theca interna cells. This might be caused by the drug delivered under physiological conditions in our study, where a homeostasis could be maintained under the mechanism of mitochondrial dynamics. In mouse T cells, mitochondrial fusion was promoted by simultaneous treatments of M1 and the fission inhibitor, Mdivi-1, suggesting the combination of multiple modulators could be necessary to change the mitochondrial networks^16^. In rats, changes in the protein levels of dynamin-related protein 1 (Drp1), the mitochondria fission protein, gradually increased from proestrus to the highest during diestrus^29^. Since the mitochondria showed an inhomogeneous distribution in cells and the ovaries were composed of growing follicles, interstitial cells, and corpus luteum in rodents, specific stains or cell isolation for effective quantitation and structural observation might be necessary to correlate the mitochondrial dynamics among the ovarian cells. As the cycle is changing faster in rodents, more frequent sampling timing for each stage of the estrous cycle might be needed.

Although the mitochondrial networks were maintained, increasing expressions of mitochondrial respiratory complexes were revealed in transcriptome analysis after the M1 treatments, suggesting that M1 could affect the ovarian cycle without completely changing the mitochondrial shape in ovarian cells. M1-induced upregulations of mitochondrial respiratory complexes were also reported in literature, suggesting a specific impact of M1 on ovarian mitochondria^12^. For steroid hormone productions, the pathways of steroid and estrogen biosynthesis were up-regulated in the M1 group. Since the concentrations of serum estradiol remain constant among the groups, the upregulations of mRNA could reflect the early responses of steroidogenic cells in a relatively short period (11 h from the first M1 injection). However, the mechanisms for the up-regulation of the pathways await more studies.

On the other hand, the downregulation of the members in progesterone-mediated oocyte maturation could reflect the inhibitions of the machinery for oocyte development in the organ, accompanied by the down-regulation of ECM molecules, remodeling enzymes, and adhesion signaling under M1 treatments. In cultured mouse ovaries, the appropriate supplies of progesterone were shown to increase follicle growth and oocyte release^30^. Our results suggested the M1 treatments could result in the stagnation of ECM remodeling and follicle growth. In our study, no pathways of apoptosis nor follicular atresia were noted. As we focused on the acute impact of M1 on ovarian tissues, more detailed studies are still needed to clarify the time course of ovarian responses to M1 treatments. Although the gene expressions could be regulated by LH in our transcriptome analysis, inhibition of progesterone synthesis and ovulation was evidenced in ex vivo cultures after M1 treatments, suggesting a direct contribution of M1 could occur in the ovaries. The expressions of the progesterone-producing enzyme, HSD3B, were evidenced in theca interna, interstitial cells, and corpus luteum as well, suggesting the multiple sources of progesterone in the mouse^31^. However, the cells under M1 inhibitions remain to be identified in further studies.

In our ex vivo study, although the inhibition of progesterone synthesis and oocyte release showed consistency with our in vivo studies, the dosage of M1 may not cause the response through identical pathways among the models. In cultured steroidogenic cells, the promotion or inhibition of steroidogenesis could depend on the dosage of the mitochondrial fission inhibitor, Mdivi-1^10^. As the detailed information on organ culture remains to be established. More experiments were necessary to confirm the detailed relationships among various models.

Mitochondria could serve as the pivot point for steroidogenic cells in reproductive systems. As the site for steroidogenic enzyme reactions, mitochondria need to get their functions balanced. In MA-10 cells, cAMP stimulation was shown to increase cellular respiration and mitochondrial membrane potential^32^. The inhibitions of electron transport, disruptions of intramitochondrial pH values, or the dissipations of mitochondrial membrane potential were sufficient to result in a decreasing progesterone production independently from the activities of mitochondrial steroidogenic enzymes^32^. While stress-induced mitochondrial biogenesis was evidenced in the Leydig cells of the rats^33^, estrous cycle changes have been linked to environmental factors^34,35^. For example, a lengthening diestrous phase could be found in the rat after seven days of hypoxia exposure^36^. To our knowledge, the mitochondrial fusion promoter, M1, was first reported to interfere with the functions of female reproductive systems in our study. Although the gene expression profiles showed a specific response to M1 in ovarian mitochondria, more linkages between mitochondria and ovarian functions are needed.

In conclusion, the effects of M1 in the late proestrus caused the vaginal smear to mimic the diestrus stage. In the ovary, M1 could lead to inhibition of ECM remodeling and cell adhesion signalings, which is accompanied by a decrease in serum LH and progesterone. The inhibited follicle growth could lead to a lower number of ovulated oocytes after the M1 treatments. Although the M1 treatments promote the expression of mitochondrial respiratory complexes, the treatment could not change the mitochondria shape in the steroidogenic cells at matured follicles. More detailed studies remain necessary to clarify the role of mitochondrial dynamics in ovarian cycles.

## Methods

### Animals

In this study, 8-week-old female ICR mice (obtained from BioLASCO) with the average weight of 25 g were housed at 25 ± 2℃, with an approximate 50–60% relative humidity and 12-h light/12-h dark cycle. Diets and water are freely accessed. Mice were acclimated for two weeks before the treatments started and placed in cages according to their experimental groups. Males were also presented in the same room to enhance the regular cycles^7^. Each cage contained 4–6 mice and was provided with enrichment (i.e. plastic tube, shredded paper, etc.). The cages were cleaned and checked every week. After the treatment, the mice were anesthetized with 2.5% avertin (0.15 mL/10 g) through intraperitoneal injection and sacrificed by cervical dislocation. All methods were also performed in accordance with ARRIVE guidelines. All the operations and the usage of animals followed the National Institutes of Health Guide for the Care and Use of Laboratory Animals (NIH Publications No.8023, advised 1978), and were approved by National Taiwan University Institutional Animal Care and Use Committee (NTU-108-EL-00112).

### M1 injection and cycle determination

The mouse estrus cycle was determined by a daily vaginal smear. To collect the cells for examinations, vaginal lavages were performed by flushing the vagina with 100 µl of saline water. The cells collected in the solution were dispersed onto a glass slide and stained using toluidine blue O (TBO)^7^. After 2 regular cycles were observed, additional smears were conducted at 8:00 on the day of the predicted proestrus. The 10 days M1 (SI-SML0629, Sigma-Aldrich, 1 mg/ml in 5% DMSO/10% Tween80/85% 1xPBS solution, 10 mg/kg BW) treatment^17^ was given after the mouse has been assured in the proestrus stage. The M1 treatment was given at 9:00 and 16:00 through IP injections. A daily vaginal smear was continued at 12:00 to observe the impact of the treatment. To observe the longer impact of the drug, 10-day treatments and vaginal smear were performed in accordance with the same treatment schedule. While to observe the acute impact of the drug, 1 day M1 treatment was performed using the same treatment and vaginal smear schedule. The serums and ovaries were collected before (8:00, P8) and after the treatments (20:00, M1 or control). The ovaries of each group were sampled for electron microscopy observation (n = 4 for each group) and for next-generation sequencing (n = 3 for each group). At the same time, the serums were collected to perform the enzyme-linked immunosorbent assays (ELISA) for the concentrations of hormones.

### ELISA

The concentration of progesterone in the serum and ovarian tissue culture medium of the mice at each stage was measured using a progesterone ELISA established in our laboratory^37^. The serum and medium estradiol were measured using Mouse Estrogen ELISA Kit (ab285291, Abcam), while the serum LH was measured using ELISA Kit for mouse luteinizing hormone (CEA441Mu, Cloud-Clone Corp., USA). All standards and samples were assayed in duplicate. The Student’s t-test was performed to identify significant differences (*P* < 0.05) between groups.

### Ultrastructural observation

After sampling, the ovaries were immediately immersed in precooled fixative containing 4% (w/v) paraformaldehyde (PFA) and 2.5% (w/v) glutaraldehyde (GA) in 0.1 M sodium phosphate buffer (PB, pH 7.3). The tissues were then dissected and trimmed into small cubes (< 1 mm^3^) in the fixative. The tissues were fixed overnight at 4 °C and further subjected to a standard protocol of post-fixation (1% Osmium tetroxide in 0.1 M PB for 90 min), dehydration, and embedding (Spurr’s medium). Semi-thin Sects. (500 nm) were cut and stained with TBO for light microscopy. The matured antral follicles without the signs of atresia were selected for further observations. Ultra-thin sections were collected on the cupper slot grids with carbon support films and the grids were stained with Reynold’s lead citrate for 4 min. The 2D images of mitochondria were acquired using a transmission electron microscope (TEM, FEI Tecnai G2 TF20 Super TWIN) operating at 120 kV. To screen the specimen, at least 3 blocks from different individuals were observed for each group. For electron tomography, serial Sects. (200 nm) through the target cells were obtained^38^. Double-tilt electron tomography was performed with an FEI Tecnai TEM operating at 200 kV. The mitochondrial volume was reconstructed, combined, and joined with eTomo^39^, while the segmentation and visualization of mitochondrial 3D structures were manually performed in Amira software (Thermo Fisher Scientific Inc.).

### RNA extraction

For each individual, both ovaries were collected and homogenized together for RNA extractions. Total RNA was extracted using the Trizol reagent (Invitrogen, CA, USA). The extraction results were evaluated in SimpliNano™—Biochrom Spectrophotometers (Biochrom, MA, USA) and Qsep 100 DNA/RNA Analyzer (BiOptic Inc., Taiwan).

### Library preparation and sequencing

For each sample, 1 μg total RNA was used to generate the sequencing library with KAPA mRNA HyperPrep Kit (KAPA Biosystems, Roche, Basel, Switzerland). Briefly, magnetic oligo-dT beads were used to purify the mRNA from total RNA. The fragmentation of captured mRNA was achieved in KAPA Fragment, Prime, and Elute Buffer (1x) according to the manual suggestions. First-strand cDNA was synthesized with the primer of the random hexamer. The cDNA-RNA hybrids were then converted into double-stranded cDNA (dscDNA) with dsDNA adapters. The library fragments were purified through the KAPA Pure Beads system (KAPA Biosystems, Roche, Basel, Switzerland) for the cDNA fragments at 300 ~ 400 bp in length. The molecules with adapter sequences at both ends were amplified in KAPA HiFi HotStart ReadyMix (KAPA Biosystems, Roche, Basel, Switzerland) with amplification primers. The PCR products were purified with the KAPA Pure Beads system. The library quality was assessed on the Qubit@ 2.0 Fluorometer (Thermo Scientific) and Agilent Bioanalyzer 2100 system. The sequencing was performed on the Illumina NovaSeq6000 platform and 150 bp paired-end reads were generated.

### Data analysis

The raw data in FASTQ format were firstly checked in FastQC and MultiQC^40^. The adaptor sequences, low-quality reads and bases were removed in Trimmomatic (v.0.38)^41^. The clean reads were aligned to the mouse reference genome (GRCm38) in HISAT2 software (v 2.1.0)^42,43^. The mapped reads for each gene were counted in FeatureCounts (v2.0.0). The DEGs and PCA between the M1 group and Control group were obtained with DESeq2 (v 1.26.0)^44,45^. To control the FDR, the *P*-adjust values were generated from the *P*-values using Benjamini and Hochberg’s approach. The DEGs with *P-*adjust below 0.05 and fold changes above 1.5 were selected. The GO and KEGG pathway analysis was conducted using clusterProfiler (v3.14.3)^46–48^. GSEA was performed with 1000 permutations to identify the activated metabolic pathways from the Gene Set Knowledgebase (GSKB)^49^.

### Ovarian tissue culture

The estrus cycle of the mice was determined using a vaginal smear, and the ovary will be collected during proestrus^50^. The ovaries were trimmed into four pieces and placed on top of 30 mm cell culture inserts in 3.5 cm dishes filled with 1 mL of culture medium. The tissues are cultured in an incubator (5% CO_2_ and 37 °C). Two types of mediums were used for the culture. Medium A contained fetal bovine serum (FBS, 5% v/v), FSH (100 mU/mL, SI-F4021, Sigma-Aldrich), LH (10 mU/mL, SI-L6420, Sigma-Aldrich), and penicillin–streptomycin (penicillin, 100 U/mL; streptomycin, 100 mg/mL) in minimum essential medium alpha (MEM-alpha, 12571063, Gibco). Medium B contained 5% FBS, 100-mU FSH, 100-mU LH, 100-U/mL penicillin, and 100 mg/mL streptomycin in MEM-alpha. Medium B was used to produce LH surges every four days, and the culture lasted for ten days (2 full cycles)^50^. For the M1 treatment, the drugs were dissolved in DMSO (1 mM) together with medium B in a final concentration of 50 μM. The medium was daily sampled to perform progesterone ELISA. The number of oocytes ovulated for each group was calculated through microscopic observations.

### Statistical Analysis

In our ELISA procedure, all standards and samples were assayed in duplicate. The Student’s t-test was performed to identify significant differences (P < 0.05) between groups. The number of ovulated oocytes were also calculated. Results are expressed as the mean ± standard error of the mean (SEM). Group results were compared using analysis of variance; P < 0.05 was considered statistically significant.

## Supplementary Information

Supplementary Information 1.

Supplementary Video 1.

Supplementary Video 2.

Supplementary Video 3.

Supplementary Video 4.

Supplementary Table 1.

Supplementary Table 2.

Supplementary Table 3.

Supplementary Table 4.

Supplementary Table 5.

## Data Availability

The datasets generated and/or analyzed during the current study are available in the Gene Expression Omnibus (GEO) repository, GSE213529.
