# Supplementary material for: The injections of mitochondrial fusion promoter M1 during proestrus disrupt the progesterone secretion and the estrous cycle in the mouse
Source: Sci Rep. 2023 Feb 10;13:2392. doi: 10.1038/s41598-023-29608-7 (PMC9918500; doi:10.1038/s41598-023-29608-7)
Supplement: Supplementary file 1 — Supplementary Information 1. [file 41598_2023_29608_MOESM1_ESM.pdf]

The Injections of Mitochondrial Fusion Promoter M1 during Proestrus Disrupt the Progesterone Secretion and the Estrous Cycle in the Mouse.

Yovita Permata Budi[1,2], Meng-Chieh Hsu[3], Yi-Chun Lin[4], Yue-Jia Lee[5], Hsin-Yi Chiu[6,7,8,9], Chih-Hsien Chiu[3], Yi-Fan Jiang[1,2*]

[1] Graduate Institute of Molecular and Comparative Pathobiology, School of Veterinary Medicine, National Taiwan University, Taipei 10617, Taiwan.

[2] School of Veterinary Medicine, National Taiwan University, Taipei 10617, Taiwan

[3] Department of Animal Science and Technology, National Taiwan University, Taipei 10617, Taiwan.

[4] Department of Animal Science, National Chung Hsing University, Taichung, 40227, Taiwan.

[5] Institute of Food Science and Technology, National Taiwan University, Taipei, 10617, Taiwan

[6] Division of Thoracic Surgery, Department of Surgery, Taipei Medical University Hospital, Taipei, 11031, Taiwan.

[7] Department of Medical Education, Taipei Medical University Hospital, Taipei, 11031, Taiwan.

[8] Department of Education and Humanities in Medicine, School of Medicine, Taipei Medical University, Taipei, 11031, Taiwan.

[9] Department of Surgery, School of Medicine, Taipei Medical University, Taipei, 11031, Taiwan.

[*]Corresponding author: Yi-Fan Jiang

E-mail: yfjiang@ntu.edu.tw

Tel: 886-2-3366-3765

Fax: 886-2-2366-1475

Mailing address: Rm. 104-1, No.1, Sec. 4, Roosevelt Road, Taipei City 10617, Taiwan

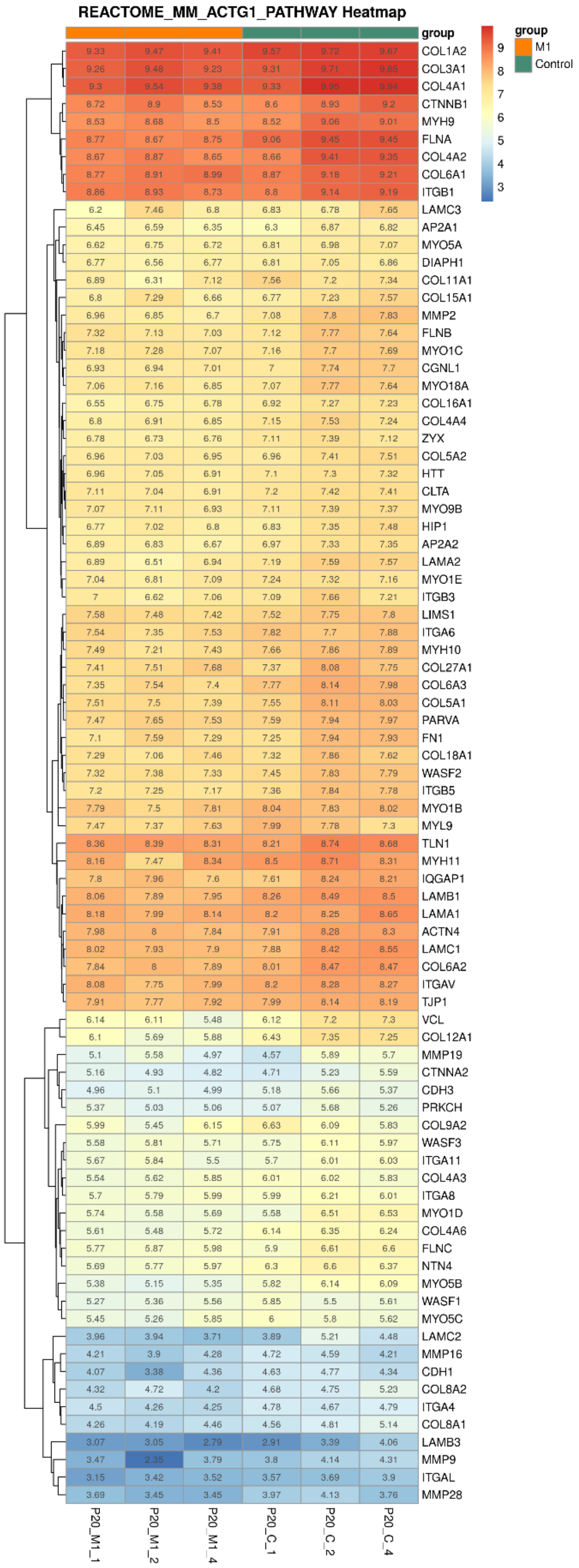

Figure S1. The heatmap of the genes in ACTG1 pathway. The pathway was obtained in gene set enrichment analysis (GSEA). The expression levels were normalized and shown as relative log expression (RLE).

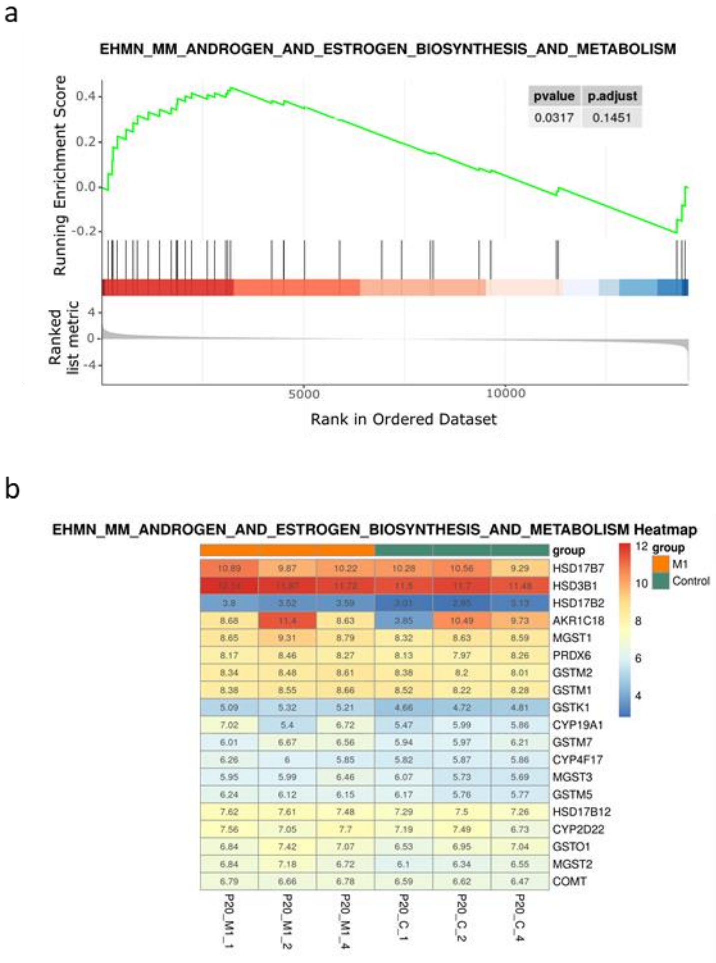

Figure S2. The GSEA plot (a) and heatmap (b) for the genes related to estrogen biosynthesis. The expression levels were normalized and shown as relative log expression (RLE).

Table S1. Mapping summary of reads.

Table S2. Differently expressed genes between M1 and Control groups.

Table S3. Gene Ontology analysis of differently expressed genes between M1 and Control groups.

Table S4. KEGG pathway analysis of differently expressed genes between M1 and Control groups.

Table S5. The list of mouse metabolic pathway from GSEA.

Movie S1. Serial-section electron tomography of the mitochondria in granulosa cells from a matured follicle in the control group. The sample was collected after the control treatment on the evening of proestrus.

Movie S2. Serial-section electron tomography of the mitochondria in granulosa cells from a matured follicle in the M1 group. The sample was collected after the M1 treatment on the evening of proestrus.

Movie S3. Serial-section electron tomography of the mitochondria in theca intrena cells from a matured follicle in the control group. The sample was collected after the control treatment on the evening of proestrus.

Movie S4. Serial-section electron tomography of the mitochondria in theca intrena cells from a matured follicle in the M1 group. The sample was collected after the M1 treatment on the evening of proestrus.
